# Low plateletcrit is associated with reduced progression: Free and overall survival in chronic lymphocytic leukemia

**DOI:** 10.5937/jomb0-39375

**Published:** 2023-03-15

**Authors:** Demircan Ozbalci, Emine Guchan Alanoglu, Eda Findos, Hande Nur Eroglu

**Affiliations:** 1 Suleyman Demirel University Research and Education Hospital, Department of Hematology, Isparta, Turkey; 2 Suleyman Demirel University Research and Education Hospital, Department of Internal Medicine, Isparta, Turkey; 3 Suleyman Demirel University Research and Education Hospital, Department of Public Health, Isparta, Turkey

**Keywords:** plateletcrit, chronic lymphocytic leukaemia, prognosis, overall survival, trombokrit, hronična limfocitna leukemija, prognoza, opšte preživljavanje

## Abstract

**Background:**

Alterations of plateletcrit and mean platelet volume (MPV) and pathogenesis of chronic lymphocytic leukaemia (CLL) have been linked to various inflammatory disorders. The prognostic impact of plateletcrit and MPV were evaluated.

**Methods:**

MPV and plateletcrit levels of both CLL and control group were compared and then in CLL patients, additional diseases, leukocyte count, platelet count, lactate dehydrogenase, Rai stage, progression-free and overall survival, mutations, if any, and chemotherapy, if any, were recorded. Then, the relationship between MPV and plateletcrit values and these parameters were evaluated in CLL patients.

**Results:**

Platelet and plateletcrit values were found to be significantly lower in CLL patients than the control group (p<0.001) for both. Plateletcrit and MPV values of patients who did not receive chemotherapy were higher than those who received chemotherapy (p=0.03, p=0.02, respectively). Being over 75 years old, plateletcrit value less than 0.1565 %, platelet level below 175 x 109/L, and leukocyte count greater than 53.5 x 109/L was found to significantly reduce overall survival. Male gender, each stage increase, plateletcrit less than 0.1565 % and leukocyte count greater than 53.5 x 109/L was related to reduce treatment-free survival in CLL patients.

**Conclusions:**

Plateletcrit can be a viable prognostic marker for defining both treatment free and overall survival.

## Abstract (sr)

**Uvod:**

Promene trombocita i srednjeg volumena trombocita (MPV) i patogeneze hronične limfocitne leukemije (CLL) su povezane sa različitim inflamatornim poremećajima. Procenjen je prognostički uticaj trombocita i MPV.

**Metode:**

Nivoi MPV-a i trombocita u HLL i kontrolnoj grupi su upoređeni, a zatim kod pacijenata sa CLL, dodatne bolesti, broj leukocita, broj trombocita, laktat dehidrogenaza, Rai stadijum, opstanak bez progresije i ukupno preživljavanje, mutacije, ako ih ima, i hemoterapija, ako ih ima, snimljeni su. Zatim je procenjen odnos između vrednosti MPV i trombocita i ovih parametara kod pacijenata sa CLL.

**Rezultati:**

Utvrđeno je da su vrednosti trombocita i trombocita značajno niže kod pacijenata sa CLL u odnosu na kontrolnu grupu (p<0,001) za oba. Vrednosti trombocita i MPV kod pacijenata koji nisu primali hemoterapiju su bile veće od onih koji su primali hemoterapiju (p=0,03, p=0,02, respektivno). Kod starijih od 75 godina, vrednost trombocita manja od 0,1565%, nivo trombocita ispod 175 x 109/L i broj leukocita veći od 53,5 x 109/L su značajno smanjili ukupno preživljavanje. Muški pol, povećanje u svakom stepenu, trombocitni krit manji od 0,1565 % i broj leukocita veći od 53,5 x 109/L bili su povezani sa smanjenjem preživljavanja bez terapije kod pacijenata sa CLL.

**Zaključak:**

Trombocitni broj može biti održiv prognostički marker za definisanje preživljavanja bez lečenja i ukupnog preživljavanja.

## Introduction

Chronic lymphocytic leukaemia (CLL) is a clonal B cell lymphoproliferative disease and is the most prevalent hematologic malignancy over 65 years. The clinical manifestations of the disease are driven by invading monoclonal b cells from bone marrow to blood, spleen, and lymph nodes [1]. Adverse prognostic factors in CLL include TP-53 mutation or deletion, older age, clinical stage, immunoglobulin heavy chain mutation and elevated serum beta-2 microglobulin concentration [2]. Fludarabine based therapies had been the backbone of the CLL treatment but in modern era, therapy heavily depends on cytogenetic analysis and in TP-53 mutated cases bruton tyrosine kinase inhibitors like ibrutinib and idelalisib and bcl-2 inhibitor venetoclax are now integrated as first line treatment of CLL [3] [4]. The results of cytogenetic analysis usually could not be available in a short period of time in most centres and there is a clear need for new, easily available, and easy to interpret biomarkers for CLL.

A common hem counter usually shows twentytwo parameters and besides from red and white blood cell indices, platelet counts and platelet indices such as mean platelet volume (MPV) and plateletcrit (PCT) can be automatically calculated. Platelets are the smallest fragment of blood components that is screened in complete blood cell count, and they are also the first component of the blood that reacts to vascular damage. When tissue damage occurs, platelets adhere to injury site and form the first haemostatic barrier for preventing haemorrhage. Besides from forming a plug, platelets also release several inflammatory cytokines such as IL-1, IL-6, and Tumour necrosis factor alpha (TNF-α) to begin the process of fibrosis [5].

MPV is the mean size of platelets calculated by hem counter and PCT is calculated automatically by (MPV x platelet count)/10000 formula. Normal median values of MPV and PCT in Turkish population were found as 7.5-12 fl and 0.239 [6] [7]. Alterations of MPV along with platelets have been linked to various diseases associated with inflammation [8]. IL-1, IL-6 and TNF-α have been shown to induce platelet synthesis [9]. In chronic inflammatory states, large platelets loaded with pro-inflammatory cytokines in circulation have been a common event, but these platelets usually migrate to inflammatory site and depleted [10]. These events usually led to significantly reduced MPV compared to normal levels [11]. PCT, as a marker bound to MPV levels, also has been shown to reflect platelet reactivity and in many studies, significant alterations of levels in chronic inflammatory states and in cancer has been shown before [12] [13].

In this study, we aimed to evaluate whether there is a significant relationship between CLL and PCT-MPV in terms of prognosis and other prognostic factors available for CLL. For this goal, a retrospective search was conducted in newly diagnosed CLL patients.

## Materials and methods

Newly diagnosed CLL patients in Suleyman Demirel University Department of Haematology from 2003 to 2020 were retrospectively added to study. For CLL diagnosis, patients were evaluated with bone marrow aspiration, biopsy and flowcytometry and who were shown as CD5, CD19 and CD23 positive with either biopsy or flowcytometry were included. Flowcytometry was carried out with Becton Dickonson FACS Canto model II, San Jose, California, United States of America. A total of 177 patients were eligible; after exclusion of patients who had incomplete data for follow-up, 110 patients have been included for the study. 254 healthy blood donors who had been applied to Blood Centre of Suleyman Demirel University Medical Hospital from 2003 to 2020 and who had no history of chronic inflammatory disease or malignancy were included for the study. Age and gender of the patients and control group were recorded. The complete blood cell count at the time of diagnosis of these patients and control group were retrospectively collected and MPV and PCT values were recorded. Complete blood cell count measurement was carried out with Beckman Coulter-Cellular Analysis System, UniCel DxH 800, Brea, California, United States of America. MPV and PCT measurements were made before transfusion. First, patients were evaluated whether there was a significant difference between the MPV and PCT values of the control group and the CLL group. Then, in CLL patients, additional diseases (systemic non-inflammatory diseases such as diabetes mellitus, hypertension, cardiovascular diseases, pulmonary diseases, neurologic diseases, psychiatric disorders), leukocyte count, platelet count, lactate dehydrogenase, Rai stage, mutations, if any, and chemotherapy, if any, were recorded. Treatment-free survival was defined as the time from the time of diagnosis to the need for the first chemotherapy, or time interval between the first and second chemotherapy in months. Overall survival was defined as time from diagnosis to death for deceased patients and for patients who were alive, the period from diagnosis to the date that the data were collected (May 2021) in months. Then, it was examined whether there was a significant relationship between MPV and PCT counts and these parameters in CLL patients.

The approval of the Ethics Committee of the Faculty of Medicine of Suleyman Demirel University dated 11.03.2021 and numbered 72867572-050.01.04-37526 was obtained for the study. Informed consent was obtained from all the participants and all study was performed in accordance with Declaration of Helsinki.

Statistics analyses were made using an IBM SPSS-26 package program. The conformity of the variables to the normal distribution was examined using visual (histogram and probability graphs) and analytical methods (Kolmogorov-Smirnov/Shapiro-Wilk tests). Descriptive analyses were given using the mean and standard deviations for normally distributed variables. Age, MPV, leukocyte count, platelet count and PCT values were compared between the control and CLL groups using Student's t test. Gender and frequency differences between the control and CLL groups were compared with the chi-square test. Then, the relationship of the parameters recorded in CLL patients with PCT and MPV values with Student's t test for additional disease, age group, gender and treatment and one-way analysis of variance for stage and mutation.

Receiver operating characteristic (ROC) curve analysis was used to determine appropriate cut-off values for clinical variables. The effects of predisposing factors on survival were calculated using the log rank test and survival rates using Kaplan-Meier survival analysis. In multivariate analysis, independent factors in predicting survival were examined using the backward LR method and Cox regression analysis using possible factors determined in previous analyses. Among the parameters with a similar effect on survival and with high correlation rates, those that were clinically significant with the model were selected. p<0.05 was considered statistically significant.

## Results

### First phase

The mean age of the CLL group was 73.7±11.9 (min 44 - max 97), and 38.2% of the patients were female. The mean follow-up period of CLL patients was 41.6±34.9 (min 1 - max 166) months. Co-morbid disease was present in 47.7% of the CLL group; these were hypertension (30 patients), diabetes mellitus (19 patients), cardiovascular diseases (13 patients) and other (16 patients). 55% of the CLL group did not receive any chemotherapy treatment and were followed without medication. No mutation was detected in 82.0% of the CLL group, 17p mutations were found in 9.0%, and other mutations in the remaining 9.0% (del 9.0% (del 13q, t [14] [15], t [11] [14], and del13q14. 31 patients were Stage 0, 30 patients were stage 1, 13 patients were stage 2, 20 patients were stage 3, and 16 patients were stage 4 respectively according to Rai classification. The mean age of the control group was 47.6±6.5 (min 40 - max 66) and 10.2% of the group were women.

When the control group was compared with the CLL group; while the mean age and leukocyte counts of CLL patients were higher than the control group (p<0.001 for both), platelet and PCT counts were found to be significantly lower than the control group (p<0.001 for both) (Table 1).

**Table 1:** Relationship between Control and CLL Group. MPV: Mean Platelet Volume, PCT: Plateletcrit, PLT: Platelet, WBC: White Blood Cell Count

Characteristic	CLL	Control	P value
Age	73.7±11.9	47.6±6.5	<0.001
Gender, Female (%)	42 (%38.2)	26 (%10.2)	<0.001
Male n (%)	68 (%61.8)	228 (%89.8)	
MPV (f/L)	8.4±1.1	8.3±0.8	0.53
PLT (x 10^9^/L)	186.1±74.0	238.8±45.6	<0.001
WBC (x 10^9^/L)	60.5±66.2	7.5±1.3	<0.001
PCT (%)	0.15±0.06	0.20±0.04	<0.001

### Second phase

In the second stage of our study, the relationship between PCT and MPV values and clinicopathologic factors in the CLL group was examined. PCT averages were found to be significantly higher in women with CLL (p=0.02). This difference was not found between MPV averages. The mean PLT was found to be 206.7±78.7 x 10^9^/L in women and 178.3±68.5 x 10^9^/L in men, and it was found to be significantly higher in women than in men (p=0.03). In addition, PCT averages differed according to the stages. PCT values of stage 4 patients were significantly lower than all stages except stage 2 (p=0.02, p=0.03, respectively). PCT values were found to be significantly lower in stage 2 patients compared to stage 0 and stage 1 patients (p<0.001). When CLL patients were grouped according to whether they received chemotherapy during follow-up, the PCT and MPV values of patients who did not receive chemotherapy were higher than those who received chemotherapy (p=0.03, p=0.02, respectively). There was no difference in PCT and MPV values according to the mutation detection status of the patients (Table 2).

**Table 2:** Relationship between PCT and MPV Values and Clinicopathological Factors. ** Significance in post hoc tests; Between Stage 4 and Stage 0, Stage 1 and Stage 3; It is between Stage 2 and Stage 0 and Stage 1.<br>CT: Chemotherapy

Characteristic		PCT			MPV		
		Mean	SS	P value	Mean	SS	P value
Gender	Female	0.17	0.07	0.02	8.38	1.24	0.99
	Male	0.14	0.05		8.38	1.01	
Age	≤75	0.15	0.06	0.48	8.53	1.14	0.15
	>75	0.16	0.06		8.23	1.06	
Stage	Stage 0	0.18	0.05	<0.001*	8.43	1.04	0.34
	Stage 1	0.18	0.06		8.46	0.95	
	Stage 2	0.13	0.04		8.76	1.02	
	Stage 3	0.14	0.06		7.98	1.07	
	Stage 4	0.08	0.05		8.30	1.50	
Treatment	CT	0.17	0.05	0.03	8.60	1.01	0.02
	No CT	0.14	0.07		8.11	1.15	
Mutation	No	0.16	0.06	0.4	8.41	1.13	0.20
	Yes (others)	0.17	0.09		8.67	0.75	
	Yes (17p)	0.13	0.08		7.78	1.00	

### Third phase

In the third phase of our study, Kaplan-Meier and Cox regression analysis was performed to evaluate the prognostic significance of MPV, PCT and other clinical and pathologic values for survival. Receiver operating characteristic (ROC) curve analysis was used to determine appropriate cut-off valuesfor clinical variables. Cut-off values determinedaccording to survival status are shown in Table 3.

**Table 3:** Cut-off Points for Clinical Variables in CLL Patients. MPV: Mean Platelet Volume, PCT: Plateletcrit, PLT: Platelet, WBC: White Blood Cell Count, LDH: Lactate Dehydrogenase

Variable	Cut-off value	AUC (%95CI)	Specificity	Sensitivity
MPV	8.55	0.581	0.643	0.562
PCT	0.1565	0.575	0.604	0.561
PLT	175	0.562	0.509	0.596
WBC	53.5	0.666	0.789	0.470
LDH	250	0.642	0.719	0.453

When the overall survival is evaluated, the mean survival time for all CLL patients was 95.7±6.8 months. In the single analyses, being over 75 years old, PCT value less than 0.1565%, platelet count below 175 x 10^9^/L, and leukocyte count greater than 53.5 x 10^9^/L were found to significantly reduce overall survival. The effects of additional disease (p=0.45) and presence of mutation (p=0.56), gender, stage, MPV and lactate dehydrogenase on overall survival were not found to be statistically significant (Table 4).

**Table 4:** Kaplan-Meier Analysis and Mean Survival Time in CLL Patients. * shows a significant linear decrease.<br>MPV: Mean Platelet Volume, PCT: Plateletcrit, PLT: Platelet, WBC: White Blood Cell Count, LDH: Lactate Dehydrogenase

Characteristic		Treatment-free survival			Overall survival		
		Mean survival	SE	p	Mean survival	SE	p
Gender	Female	79.5	9.3	0.01	98.1	10.6	0.69
	Male	43.7	5.9		93.4	8.9	
Age	≤75	47.4	6.4	0.14	120.4	10.3	0.01
	>75	66.6	8.1		76.4	7.7	
Stage	Stage 0	81.1	9.6	<0.001*	111.8	11.9	0.05
	Stage 1	81.1	11.4		97.3	9.8	
	Stage 2	63.2	15.4		70.2	13.0	
	Stage 3	24.4	5.5		61.0	12.6	
	Stage 4	24.9	6.5		100.8	19.3	
MPV	≤8.55	57.8	7.4	0.60	88.2	8.4	0.24
	>8.55	53.0	7.0		105.7	11.2	
PCT	≤0.1565	45.1	6.0	0.03	82.8	9.2	0.04
		71.6	8.7		102.7	7.9	
PLT	≤175	44.9	6.5	0.08	77.2	10.2	0.01
	>175	66.4	7.7		108.2	8.5	
WBC	≤53.5	71.3	7.1	<0.001	112.2	8.3	<0.001
	>53.5	33.0	6.7		61.8	8.7	
LDH	≤250	67.3	7.6	0.08	106.7	9.1	0.13
	>250	45.0	6.8		81.7	9.4	
**Total**		**58.7**	**5.6**		**95.7**	**6.8**	

In the evaluation of treatment-free survival, the duration of the CLL group without treatment was 58.7±5.6 months. Male gender, each stage increase (linear significance), PCT value less than 0.1565% and leukocyte count greater than 53.5 x 10^9^/L were found to be prognostic factors that reduced treatment-free survival in CLL patients. Co-morbidity (p= 0.70) and presence of mutation (p= 0.20) did not have any effect on treatment-free survival as well as overall survival (Table 4).

Finally, a Cox regression analysis was performed by including factors that were significant in univariate analyses, whose p value was close to the type 1 error value (with a cut off value of p:0.25) and whose correlation matrix of regression coefficients value was below 0.6. In multivariate analysis, male gender (HR, 2.27; p = 0.016), leukocytes>53.5 x 10^9^/L (HR, 2.03; p = 0.016), stage (with each stage increase; HR, 1.66; p<0.001), presence of mutation (none, the presence of other mutations, the presence of 17p mutations) were defined as statistically significant predictive factors that reduced treatment-free survival in each category increment, HR, 1.85; p = 0.003). In the multivariate analysis for overall survival in CLL patients; age>75 (HR, 2.44; p = 0.003), leukocyte>53.5 x 10^9^/L (HR, 2.57; p = 0.001) were defined as statistically significant predictive factors that reduced overall survival (Table 5).

**Table 5:** Multivariate Survival Analysis in CLL Patients. * At each linear increment. **linear none, there is other mutation, there is 17p mutation in each category increase.<br>Method: Backward Stepwise (likelihood ratio); Treatment-free survival; –2 Log likelihood: 396,966; Omnibus test of model coefficients: p=0.000. Overall survival; –2 Log likelihood: 425,874; Omnibus test of model coefficients: p=0.000<br><br>PCT: Plateletcrit, WBC: White Blood Cell Count

	Treatment-free Survival			Overall Survival		
	Hazard Ratio	95% CI	p	Hazard Ratio	95% CI	p
Gender						
Female	1					
Male	2.278	1.169–4.439	0.016			
Age						
≤75				1		
>75				2.436	1.685	0.003
Stage Linear*	1.662	1.341–2.060	<0.001			
Mutation**	1.845	1.227–2.773	0.003			
PCT						
≤0.1565				1.685	0.955–2.973	0.072
>0.1565				1		
WBC						
≤53.5	1			1		
>53.5	2.027	1.139–3.608	0.016	2.568	1.480–4.456	0.001

## Discussion

In this study, low PCT was associated with reduced treatment free and overall survival and had prognostic value in CLL. PCT and MPV were also related to Rai stage change and need for therapy. Stage 4 patients had significantly low MPV and PCT levels and the need to initiate therapy was more consistent in low PCT and MPV patients compared to high group.

In literature, conflicting results were seen for PCT and malignancy connection; malignancies were generally associated with high plateletcrit and MPV levels. Sahin et al. [12], found that, PCT was significantly elevated in lung cancer compared to healthy controls. Hur et al. [13] showed that high plateletcrit was associated with poor outcome in non-small cell cancer. Zhu et al. [14] found that PCT levels were higher in colorectal cancer patients and PCT levels were significantly associated with tumour stage, size, and vascular invasion. Also, high PCT and MPV levels were reported to be associated with endometrial cancer [16]. Contrary to those reports, Oncel et al. [17], showed that, PCT levels were significantly lower in patients with metastatic lung cancer. Also, in osteosarcoma, PCT was not associated with disease activity, stage, or prognosis [18]. These contradicting results might be due to different origins and inflammatory pathways related to that specific disease.

PCT as well as MPV had been shown to significantly link to inflammation in several studies, high plateletcrit levels were seen in various active inflammatory states [15] [19] [20] [21]. The pathogenesis of CLL was also linked to inflammation; interleukin 1 (IL-1), interleukin 6 (IL-6) and tumour necrosis factor alpha (TNF-α) has been shown to be key cytokines affecting both inflammation and CLL progression [22]. These cytokines lead to activation of Janus kinase pathway resulting activation of multiple oncogenes [23]. TNF-α has been linked to proliferation of B cells as well as activating NF-B, a proinflammatory pathway and IL-6 was shown both to increase survival of CLL cells and proinflammatory miR-21 [24] [25] [26] [27]. In that point, PCT also might be elevated in CLL similarly due to this inflammatory pathogenesis but, we found that low PCT was associated with adverse outcome. Chronic, not acute, inflammation seemed to dominate CLL; many patients were diagnosed years after the beginning of CLL due to natural course of disease. In chronic inflammation, large platelets were found to be released from bone marrow induced by pro-inflammatory cytokines, then later, these platelets were depleted on ongoing inflammation and a significant drop on MPV levels could be seen in patients with chronic inflammation [10] [11]. Also, chronic proinflammatory cytokine release could lead to release of small therefore low-MPV platelets so, MPV and PCT levels could be found to be decreased [28]. These mechanisms should be responsible for low PCT associated poor outcome in CLL. In diffuse large B cell lymphoma, two studies have shown that low MPV was associated with worse prognosis [29] [30]. Masternak et al, found that low MPV was associated with adverse outcome in CLL; patients who need treatment had lower MPV levels compared to patients who did not need any [28]. Also, low MPV was associated with shorter time for treatment and a tendency to lower MPV levels was seen as Rai stage advances besides from stage IV in this study. Similarly, in our study, PCT less than 0.1565% was significantly associated with reduced treatment free and overall survival so we can conclude that our findings seemed to be compatible to literature.

There were several drawbacks in our study. The design was a retrospective one; a prospective study on this topic could be clearly preferred. The mean age of CLL patients were significantly higher and platelet levels were significantly lower compared to controls. The median age for CLL diagnosis is 65 years old so finding healthy age-matched healthy individuals was nearly impossible also, lower platelets in CLL patients could also be attributed to nature of the disease. Besides from that, this study is valuable in many ways; this is the first study to show that PCT levels were significantly lower in CLL patients, and it was highlighted for the first time that low PCT levels were significantly associated with worse overall survival and shorter treatment free survival in CLL albeit in univariate analysis.

In conclusion, this is the first study to show that PCT levels in CLL patients were significantly lower compared to control group. PCT levels were significantly elevated in women compared to men with CLL. PCT and MPV levels were significantly lower in CLL patients in need for treatment compared to those without. Rai stage II and IV patients had significantly low PCT levels compared to other stages and PCT levels lower than 0.1595% were significantly associated with both worse treatment-free and overall survival. Prospective and, if it can be arranged, age-matched controlled studies are needed to validate those results.

## Dodatak

### Funding

The authors did not receive support from any organization for the submitted work.

### Acknowledges

The authors appreciate the efforts of Prof. Dr. Hasan Yasan for the study.

### Data availability

All study data are available upon request from corresponding author Demircan Ozbalci.

### Author Contributions

DO: Conceptualization, Methodology, Writing - original draft preparation, Writing - review and editing, Supervision, EGA: Writing - review and editing, Supervision, EF: Writing - review and editing, Resources, HNE: Formal analysis and investigation, Writing - review and editing.

### Conflict of interest statement

All the authors declare that they have no conflict of interest in this work.

### List of non-standard abbreviations:

CLL, chronic lymphocytic leukaemia;<br>MPV, mean platelet volume;<br>PCT, plateletcrit;<br>TNF-α, tumour necrosis factor alpha.
